# Vitamin D Correction Down-Regulates Serum Amyloid P Component Levels in Vitamin D Deficient Arab Adults: A Single-Arm Trial

**DOI:** 10.3390/nu12092880

**Published:** 2020-09-21

**Authors:** Osama E. Amer, Malak N. K. Khattak, Abdullah M. Alnaami, Naji J. Aljohani, Nasser M. Al-Daghri

**Affiliations:** 1 Biochemistry Department, College of Science, King Saud University, Riyadh 11451, Saudi Arabia; osamaemam@gmail.com (O.E.A.); malaknawaz@yahoo.com (M.N.K.K.); aalnaami@yahoo.com (A.M.A.); 2 Specialized Diabetes and Endocrine Center, King Fahad Medical City, Riyadh 11525, Saudi Arabia; najij@hotmail.com

**Keywords:** vitamin D, SAP, amyloidosis, Arab, vitamin D supplementation

## Abstract

Vitamin D (VD) has been observed to have anti-inflammatory properties. However, the effects of VD supplementation on the serum amyloid P component (SAP) has not been established. This study aimed to investigate the effect of VD supplementation on serum SAP levels in Arab adults. A total of 155 VD-deficient adult Saudis (56 males and 99 females) were recruited in this non-randomized, 6-month, single-arm trial. The intervention was as follows; cholecalciferol 50,000 international units (IU) every week for the first 2 months, followed by 50,000 twice a month for the next two months, and for the last two months, 1000 IU daily. Serum 25(OH)D, SAP, C-reactive protein (CRP), lipid profile, and glucose were assessed at baseline and post-intervention. At post-intervention, VD levels were significantly increased, while SAP levels significantly decreased in all study participants. Remarkably, this reduction in SAP was more significant in males than females after stratification. SAP was inversely correlated with VD overall (r = −0.17, *p* < 0.05), and only in males (r = −0.27, *p* < 0.05) after stratification according to sex after 6 months of VD supplementation. Such a relationship was not observed at baseline. VD supplementation can favorably modulate serum SAP concentrations in Arab adults, particularly in males.

## 1. Introduction

Vitamin D (VD) is a fat-soluble secosteroid hormone, having both autocrine and endocrine roles [1]. While the main roles of VD include calcium homeostasis and bone metabolism [2,3], the presence of vitamin D receptors (VDR) in major cell types of the body gives it multiple extra-skeletal functions, one of which is modulation of inflammatory pathways [4,5].

The anti-inflammatory and immune-modulating properties of vitamin D (VD) are well-established [6]. Multiple studies consistently reveal the beneficial effects of VD supplementation in terms of increasing levels of anti-inflammatory markers and decreasing the production of inflammatory cytokines [7,8,9]. In a recent systematic review and meta-analysis involving 10 clinical trials and 924 participants, Chen and colleagues concluded that supplementation with VD can decrease C-reactive protein (CRP) levels, a well-known acute-phase inflammatory marker predictive of cardiovascular events, by as much as 2.21 mg/L [10]. Other inflammatory markers have been investigated, such as (IL)-10, IL-6 and TNF-α, all of which have been observed to be significantly associated with varying levels of 25(OH)D status [8,11,12].

Another acute-phase inflammation-induced protein is the serum amyloid P component (SAP), not to be confused with serum amyloid protein. Together, SAP and CRP are the short pentraxins chiefly produced by hepatocytes [13]. In humans, SAP contributes to host defense, either via opsonins or through complement activation. In a calcium-dependent way, SAP binds to several lipoprotein ligands, which suggests that this process could have significant inferences in amyloidosis and atherosclerosis in humans. Moreover, many studies support the fact that SAP has a significant role in inflammatory regulation. [14]. Significantly, SAP and CRP share structural characteristics (being organized in five identical subunits arranged in a pentameric radial symmetry) and biological functions, including activation of the complement system and pathogen recognition [13]. In calcium-free conditions, SAP pentamers physically interact with CRP pentamers to form very stable mixed decamers [15], which could have functional consequences on inflammation activation [16]. In a nested case-control proteomic analysis study, sera from 60 obese women with gestational diabetes mellitus (GDM) identified three candidate predictors of GDM: SAP, afamin, and vitronectin [17]. Lastly, for cardiovascular disease (CVD), SAP is considered as a valuable biomarker, as it contributes to CVD pathogenesis through modulating innate immunity and inflammation [18].

We hypothesize that improving VD status can favorably regulate SAP activity. In this single-arm trial, we aim to evaluate for the first time the effects of vitamin D supplementation on serum SAP levels in Saudi adults with VD deficiency.

## 2. Methods

### 2.1. Study Design and Participants

**Figure 1:**
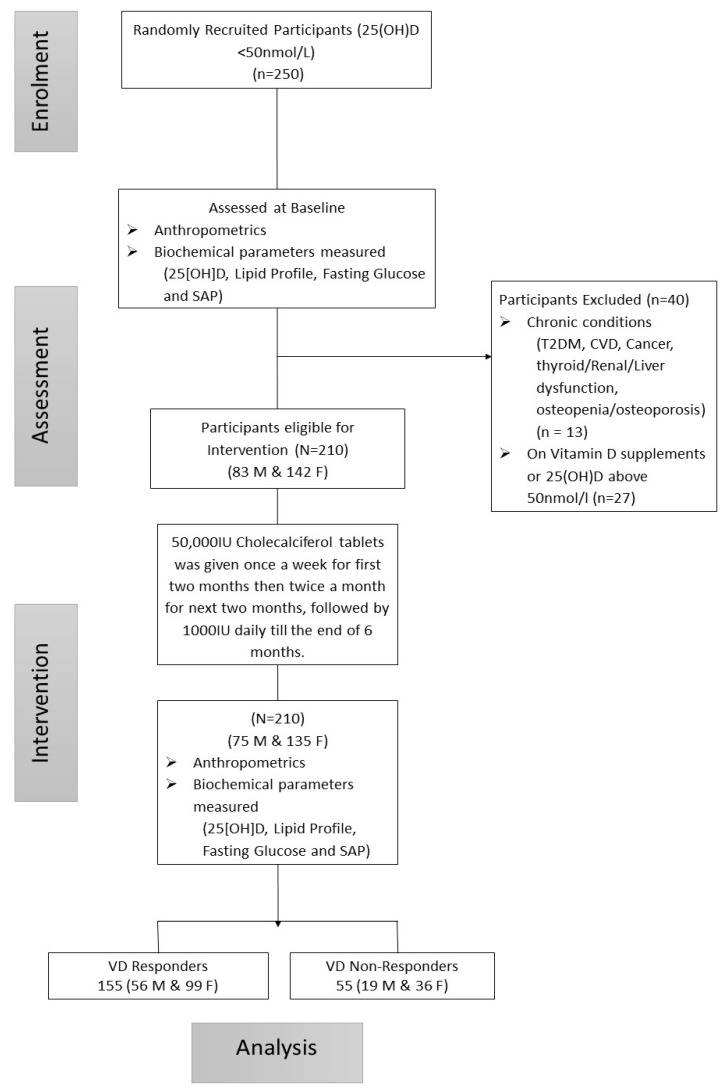
Participant flow chart.

In this 6-month, single-arm trial, a total of 250 overweight Saudi adult males and females aged 30–50 years with 25(OH) D deficiency (<50 nmol/L) were selected randomly from the Vitamin D School database of the Chair for Biomarkers of Chronic Diseases (CBCD) in King Saud University (KSU, Riyadh, KSA). In brief, this database was taken from a capital-wide, multi-center observational study done in primary and secondary schools in Riyadh, Saudi Arabia [19,20]. Written informed consent was obtained from all participants before enrolment. A generalized questionnaire was taken from all participants, including demographic information, and present and past medical history. This intervention study was conducted from December 2015 to May 2016 (the cold season in Riyadh). The present study was part of a bigger project registered in the Saudi Clinical Trials Registry (SCTR) (E1-15-1667) Riyadh, Saudi Arabia, and was approved by the Scientific Research Ethics Committee at King Fahd Medical City (16-018), Riyadh, Saudi Arabia. Exclusion criteria were as follows: those with chronic clinical conditions (cancer, cardiovascular diseases (CVD), T2DM, osteopenia/osteoporosis, gastrointestinal disease, liver and renal dysfunction, and thyroid conditions), on VD supplementation or any medication and those with baseline 25(OH)D ≥ 50 nmol/L. Out of 250 enrolled participants, 13 were excluded for having one of the mentioned conditions, and another 27 for having basal 25(OH)D levels ≥50 nmol/L. Overall, 210 participants (75 males and 135 females) were able to complete the study (Figure 1).

### 2.2. Anthropometry and Biochemical Assessments

Anthropometrics which were determined included height (rounded off to the nearest 0.5 cm), weight (rounded off to the nearest 0.1 kg), waist and hip circumference (centimeters), and mean blood pressure (systolic and diastolic in mmHg) (average of two readings). Body mass index (BMI) was calculated as weight in kilograms divided by height in square meters. Fasting blood samples were collected and transferred immediately to a non-heparinized tube for centrifugation. Fasting glucose, lipid profile, were measured using a chemical analyzer (Konelab, Espoo, Finland). Serum 25(OH)D was measured by using commercially available kits using Roche Elecsys Modular Analytics Cobas e411 utilizing electrochemiluminescence immunoassay (Roche Diagnostics, Mannheim, Germany). Serum levels of SAP and CRP were measured using ELISA kits (Abcam^®^, Cambridge, UK and R & D SYSTEMS^®^, Minneapolis, MN, USA, respectively) following manufacturers’ instructions. To minimize inter-assay variability, all samples were analyzed simultaneously and the actual variations were well within the inter- and intra-assay ranges. All measurements were done at baseline and post-intervention.

### 2.3. Intervention

VD supplementation was given to all participants in the following manner: (1) 50,000 IU cholecalciferol tablets given once a week for the first two months (VitaD50000; Synergy pharma, Dubai, UAE); (2) 50,000 IU cholecalciferol tablets twice a month for the next two months; and (3) 1000 IU daily (VitaD1000; Synergy pharma, Dubai, UAE) in the last 2 months as maintenance. The Short Message Service (SMS) was used to encourage participants to take their recommended doses of VD. For compliance, all participants had to return blister packs to quantify unconsumed tablets every month before a fresh refill was given. Intervention doses were according to the national and regional recommendations on management of vitamin D deficiency [21,22]. For stratification purposes, post-intervention responders were defined as those who achieved 25 (OH)D levels above 50 nmol/L, while non-responders were those who did not achieve 25 (OH)D levels > 50 nmol/L.

### 2.4. Statistical Analysis

Data were analyzed using the Statistical Package for Social Sciences (SPSS 22.0, SPSS, Inc., Chicago, IL, USA). Continuous data were presented as mean ± standard deviation (SD) for normal variables and non-normal variables were presented in median (first and third) percentiles. All categorical variables were presented as frequency and percentages. The Independent *T*-test and Mann-Whitney U test were used to compare baseline differences between normal and non-normal variables, respectively. Bonferroni correction was done for multiple comparisons at baseline to minimize type 1 error. The paired *T* test and Wilcoxon sign rank test were performed to check the mean and median differences at baseline and after intervention. Pearson’s and Spearman’s correlation were performed to determine associations of SAP with other parameters. The Bonferroni-adjusted *p*-value for baseline comparisons was *p* < 0.0038. A *p*-value < 0.05 was considered significant for the rest of the analysis.

## 3. Results

A total of 210 (75 males and 135 females) Saudi adults deficient in vitamin D were included in this 6-month interventional study. Table 1 shows the clinical characteristics of participants before and after intervention for responders and non-responders to VD supplementation. At baseline and using the Bonferroni-corrected *p*-value, responders were significantly older than non-responders (*p* = 0.007). Similarly, WHR measures were significantly higher in responders than non-responders (*p* < 0.001). Baseline BMI, blood pressure, and other parameters were not significantly different between groups.

Post-intervention, 25(OH)D and HDL-cholesterol levels significantly increased after 6 months (*p*-values < 0.001 and 0.007, respectively) in the responders group. In contrast, SAP levels significantly decreased post-intervention (*p* = 0.002), as well as CRP levels (*p* = 0.014). No significant changes were observed in other parameters. Among non-responders, no changes in 25(OH)D levels were observed post-intervention. The same non-significance was observed for SAP and CRP levels. Total cholesterol, HDL-cholesterol, and triglycerides all significantly increased after intervention (*p*-values = 0.006, 0.003 and 0.02, respectively). For the rest of the other parameters, no significant differences were observed (Table 1).

Table 2 shows the between-group comparisons of both responders and non-responders. Serum 25(OH)D increased over time, and this was clinically significant in favor of the responders, even after adjusting for age and BMI (*p* < 0.001). A clinically significant decrease in SAP levels was observed over time, again in favor of responders, and this effect remained significant even after adjusting for age and BMI (*p* = 0.001).

Table 3 shows comparisons of responders’ characteristics pre- and post-intervention according to sex. Levels of 25(OH)D significantly increased over time in both sexes (*p* < 0.001). Similarly, HDL was significantly increased in both sexes. SAP was significantly decreased over time in both sexes; remarkably, this reduction in SAP was more significant in males [55.7 (31.2–78.4) vs. 57.3 (27.7–100.9), *p* = 0.01] than in females [28.9 (1.4–62.4) vs. 38.4 (1.3–74.1), *p* = 0.046]. Conversely, CRP was significantly reduced post-intervention in females [7.8 (4.4–32.4) vs. 22.2 (3.9–61.6), *p* = 0.036], but no significant difference was observed in males. No reduction in glucose levels was observed in both sexes; contrary to what was expected, glucose had a significant increase in males [5.87 ± 0.9 vs. 5.61 ± 0.9, *p* = 0.029], with no significant difference observed in females.

Table 4 shows the bivariate correlation coefficients of SAP with other study parameters in responder participants at baseline, where SAP had a significantly positive relationship with systolic BP (r = 0.20, *p* < 0.05) and diastolic BP (r = 0.33, *p* < 0.01) in our study participants overall. This relationship was observed for diastolic BP only in males (r = 0.30, *p* < 0.05) after stratification according to sex. At baseline, SAP also had a significant inverse correlation with HDL-cholesterol (r = −0.30, *p* < 0.01). Overall, this clinically significant inverse correlation persisted in females (r = −0.37, *p* < 0.01) but not in males after stratification according to sex. In addition, SAP had a significantly positive correlation with glucose (r = 0.32, *p* < 0.05) in males at baseline, as well as with CRP overall and in both sexes (*p* < 0.001).

Post-intervention, SAP was inversely correlated with VD overall (r = −0.17, *p* < 0.05) and only in males (r = −0.27, *p* < 0.05) after stratification according to sex, whereas such a relationship was not observed at baseline. Triglycerides had a significant positive correlation with SAP only in females post-intervention (r = 0.23, *p* < 0.05) but not in males.

Table 5 shows the delta change correlation analysis between SAP and other parameters. Overall, there was a significant inverse relationship between Δ SAP and Δ HDL (r = −0.30, *p* < 0.01), and it was positively correlated with Δ CRP (r = 0.28; *p* < 0.01) in our study population. After stratification according to sex, Δ SAP was inversely correlated with Δ HDL (r= −0.31; *p* < 0.05) and Δ triglycerides (r = −0.27; *p* < 0.05) only in males.

Table 6 shows the responders’ characteristics pre- and post-intervention using the SAP cut-off values, a normal reference interval for serum SAP concentration, for both sexes (males; 32 mg/L and females; 24 mg/L) [23]. Of the participants, 98 (42 males and 56 females) had high values of serum SAP above referenced normal levels. Over time, 25(OH)D significantly increased in both sexes (*p* < 0.001). Remarkably, post-supplementation with VD, the reduction in SAP serum levels was more significant in this sub-group compared to the main group in both sexes (in males, −9.5 (−33.9–7.1), *p* = 0.007 vs. −1.75 (−21.7–7.4), *p* = 0.011; in females, −13.9 (−33.3–2.2), *p* < 0.001 vs. −0.57 (−16.5–1.2), *p* = 0.046).

## 4. Discussion

The present interventional study is, to our knowledge, the first to show a clinically significant reduction in serum SAP levels after 6 months of VD correction. Remarkably, the post-intervention reduction in serum SAP levels was even more significant than without applying the cut-off values. At baseline, SAP levels were inversely correlated with cardiometabolic factors, such as BMI and HDL-cholesterol, and positively correlated with blood pressure, with no association between VD and SAP. However, at post-intervention, our results showed a significant inverse correlation between SAP and VD among responders, and this significant correlation persisted in males after stratification for sex.

The link between SAD and VD based on the present results is most likely tied to their associations with cardiometabolic factors. SAP has a key role in innate immunity and cardiometabolism [24]. Furthermore, like VD, it is also directly influenced by calcium [25]. In a calcium-dependent manner, SAP binds to many different lipoprotein ligands, and this can have a significant contribution in the progression of amyloidosis and atherosclerosis [26,27]. In fact, it has been found in the plaques of advanced human atherosclerosis and is proposed to have an active role in atherogenesis [28]. Previous studies indicated a significant increase in SAP levels in the early phase of post-acute myocardial infarction [29]. Furthermore, SAP deficiency prevents the atherosclerotic process [30] and other pathological processes, such as fibrosis, hypercoagulation, and inflammation [24,31]. Lastly, pentraxins including SAP have been demonstrated to be involved in obesity and other states of a chronic low-grade inflammatory [32]. Hence, VD supplementation can reduce the cardiovascular risk associated to overweight and obesity by reducing the pro-inflammatory pentraxin SAP.

Another highlight of the present study is the significant inverse correlation post-supplementation between SAP and VD only among male responders, which highlights sex-specific extra-skeletal properties of VD correction. Previously, we found that VD deficiency and its association with cardio-metabolic risk factors were mostly limited to males, in a study which involved more than 3000 Saudi adolescents and adults. This led us to believe that correction of VD status could prove more beneficial to men than women, at least in terms of extra-skeletal benefits [20]. One explanation that we have also recently documented at the proteomic level is that the conversion of 25(OH)D to its active form, 1,25(OH)D2, is higher in men than women, and this can be linked to the sex hormone metabolism [33].

Lastly, it is worthy to discuss that the primary grouping variable used in the present study to elicit differences between circulating SAP was the participants’ response to vitamin D supplementation. Despite monitoring all participants for compliance and adherence, it was anticipated that some will not be able to achieve full vitamin D sufficiency despite large boluses of vitamin D. The failure to achieve full vitamin D correction despite above-average supplementation has been a consistent dilemma in Saudi Arabia and the rest of the region, and this has been fully acknowledged by national and regional experts, prompting vitamin D guidelines unique to the Middle-Eastern region and the Gulf Cooperation Council (GCC) countries in particular (21, 22). A recent genetic study within the Saudi community that could partially explain the non-responsiveness to exogenous vitamin D sources are the variants in vitamin D binding proteins (rs7041 and rs4588), carriers of which are three to 12 times more likely to be non-responders to vitamin D treatment [34].

The authors acknowledge some limitations. First, we used the non-responders as our comparator group, since we wanted to clearly delineate that the modest but significant changes in circulating SAP was associated with acute changes in vitamin D status brought about by a favorable response to vitamin D supplementation. Furthermore, since VD deficiency is very common in Saudi Arabia, the use of a true control group (without supplementation) is inappropriate, given that the inclusion criteria are participants with VD deficiency. Whether the present results will be the same using a control group remains to be investigated. Second, important factors influencing VD status were not measured in the current study, such as sunlight exposure, season, and outdoor physical activity, and as such, essential adjustments were not carried out. Nevertheless, this is the first study of its kind to investigate the effects of VD supplementation on SAP levels.

## 5. Conclusions

This is the first study to demonstrate the inverse relationship between serum VD and SAP. The present study showed that VD correction can significantly reduce serum SAP concentrations, particularly in male participants. As such, one of the cardiometabolic benefits of VD supplementation is through modulation of serum SAP levels, which can decrease risk for atherosclerosis, plaque formation, and multi-organ fibrosis. Further investigations are needed to determine whether prolonged states of vitamin D sufficiency can reverse atherosclerotic and fibrotic conditions through normalcy of SAP levels.

## Figures and Tables

**Table 1:** Clinical characteristics of study participants at baseline and after 6-month intervention.

Parameters	Responders				Non-Responders				* *p*-Value
	Before	After	Change	*p*-Value	Before	After	Change	*p*-Value	
N (M/F)	155 (56/99)				55 (19/36)				
Age (year)	39.9 ± 10.6				34.6 ± 11.2				0.007
BMI (kg/m^2^)	29.2 ± 4.9				27.1 ± 5.1				0.98
WHR	0.94 ± 0.13				0.87 ± 0.11				<0.001
SBP (mmHg)	124.5 ± 13.6				125.3 ± 15.4				0.73
DBP (mmHg)	77.4 ± 8.9				78.4 ± 9.5				0.50
TC (mmol/L)	5.15 ± 1.2	5.16 ± 1.2	0.02 (−0.16–0.19)	0.86	5.10 ± 0.9	5.51 ±1.2	0.44 (0.13–0.8)	0.006	0.67
HDL-C (mmol/L)	1.04 ± 0.4	1.16 ± 0.4	0.11 (0.03–0.19)	0.007	1.1 ± 0.5	1.3 ± 0.4	0.22 (0.08–0.37)	0.003	0.88
LDL-C (mmol/L)	3.26 ± 0.9	3.15 ± 1.0	−0.11 (−0.26–0.05)	0.17	3.3 ± 0.8	3.4 ± 0.9	0.14 (−0.14–0.41)	0.33	0.79
TG (mmol/L)	1.72 ± 1.1	1.75 ± 1.0	0.03 (−0.13–0.19)	0.70	1.4 ± 0.8	1.7 ± 0.9	0.29 (0.04–0.54)	0.02	0.08
25(OH)D (nmol/L)	31.7 ± 11.7	63.8 ± 19.8	32.1 (29.0–35.2)	<0.001	31.9 ± 15.3	29.1 ± 12.4	−2.8 (−6.2–0.7)	0.11	0.60
Glucose (mmol/L)	5.48 ± 0.9	5.55 ± 0.9	0.07 (−0.10–0.23)	0.43	5.4 ± 0.9	5.5 ± 0.8	0.13 (−0.17–0.44)	0.39	0.93
SAP (mg/L)	44.9 (3.1–84.8)	41.2 (2.5–69.1)	−2.8 (−18.2–1.74)	0.002	40.0 (16.9–57.6)	42.4 (21.7–67.3)	1.42(−5.6–8.4)	0.29	0.44
CRP (µg/mL)	22.8 (4.5–53.5)	13.5 (4.9–34.5)	−8.2 (−16.6–2.24)	0.014	39.9(14.9–75.9)	34.7 (8.0–80.4)	−3.5 (−20.1–6.9)	0.74	0.038

Note: Data presented as mean ± SD for normal and median (first–third) percentiles for non-normal variables. BMI—body mass index; WHR—waist-hip ratio; SBP—systolic blood pressure; DBP—diastolic blood pressure; TC—total cholesterol; TG—triglycerides; * Bonferroni corrected *p*-value applied to actual *p*-values; significant at *p* < 0.0038. M: Male; F: Female; SAP: serum amyloid P component; CRP: C-reactive protein.

**Table 2:** Between-group comparisons in 25(OH)D and Serum Amyloid P Component.

Parameters	25(OH) D (nmol/L)		Group Effect	Group Effect (Adjusted)
	Responders	Non-Responders		
Baseline	31.7 ± 11.7	31.9 ± 15.3	<0.001	<0.001
6 month	63.8 ± 19.8	29.1 ± 12.4		
Mean Difference	32.1 (29.0–35.2)	−2.8 (−6.2–0.7)		
Change (%)	103%	8.8%		
Time effect	<0.001			
Time effect (adjusted)	<0.001			
	**Serum Amyloid P Component (mg/L)**			
	**Responders**	**Non-Responders**	0.004	0.001
Baseline	44.9 (3.1–84.8)	40.0 (16.9–57.6)		
6 month	41.2 (2.5–69.1)	42.4 (21.7–67.3)		
Mean Difference	−2.8 (−18.2–1.74)	1.42(−5.6–8.4)		
Change (%)	6.2%	3.6%		
Time effect	0.017			
Time effect (adjusted)	<0.001			

Note: Data presented as mean ± standard deviation, median (first and 75th) percentiles, and mean and median change (95% CI); adjusted for age and BMI; significant at *p* < 0.05.

**Table 3:** Clinical characteristics of responders at baseline and after 6-month intervention according to sex.

Parameters	Male				Female			
N (M/F)	56				99			
Age (year)	41.9 ± 9.6				37.9 ± 11.0			
BMI (kg/m^2^)	28.4 ± 3.8				29.6 ± 5.4			
WHR	0.98 ± 0.10				0.92 ± 0.15			
SBP (mmHg)	130.4 ± 11.5				120.3 ± 13.6			
DBP (mmHg)	81.2 ± 7.1				74.8 ± 9.1			
	**Baseline**	**6-Month**	**Δ**	***p*-Value**	**Baseline**	**6-Month**	**Δ**	***p*-Value**
TC (mmol/L)	5.02 ± 1.2	5.11 ± 1.4	0.09 (−0.19–0.37)	0.52	5.22 ± 1.2	5.20 ± 1.1	−0.03 (−0.24–0.19)	0.79
HDL-C (mmol/L)	0.94 ± 0.3	1.02 ± 0.4	0.08 (0.001–0.16)	0.05	1.11 ± 0.5	1.24 ± 0.4	0.13 (0.01–0.25)	0.03
LDL-C (mmol/L)	3.16 ± 0.8	3.06 ± 1.04	−0.10 (−0.4–0.19)	0.50	3.32 ± 0.9	3.20 ± 0.9	−0.12 (−0.31–0.07)	0.23
TG (mmol/L)	1.89 ± 1.4	1.97 ± 1.2	0.08 (0.001–0.16)	0.54	1.61 ± 0.9	1.62 ± 0.8	0.003 (−0.19–0.20)	0.98
25(OH)D (nmol/L)	34.5 ± 10.3	64.6 ± 17.8	30.1 (24.9–35.3)	<0.001	30.1 ± 12.2	63.3 ± 20.9	33.2 (29.3–37.1)	<0.001
Glucose (mmol/L)	5.61 ± 0.9	5.87 ± 0.9	0.25 (0.03–0.47)	0.029	5.41 ± 1.0	5.38 ± 0.8	−0.03 (−0.26–0.20)	0.80
SAP (mg/L)	57.3 (27.7–100.9)	55.7 (31.2–78.4)	−1.75 (−21.7–7.4)	0.011	38.4 (1.3–74.1)	28.9 (1.4–62.4)	−0.57 (−16.5–1.2)	0.046
CRP (µg/mL)	26.8 (4.7–48.5)	22.1 (5.2–34.8)	−2.42 (−10.9–3.6)	0.20	22.2 (3.9–61.6)	7.8 (4.4–32.4)	−0.10 (−28.9–2.20)	0.036

Note: Data presented as mean ± SD for normal, and median (1st–3rd) percentiles for non-normal variables. BMI—body mass index; WHR—waist-hip ratio; SBP—systolic blood pressure; DBP—diastolic blood pressure; TC—total cholesterol; TG—triglycerides; Significant at *p* < 0.05.

**Table 4:** Bivariate associations of SAP among responders at baseline and after 6-month intervention.

Parameters	Baseline			6-Month		
	All	Males	Females	All	Males	Females
Age (year)	0.08	−0.04	0.04			
BMI (kg/m^2^)	−0.03	−0.05	0.04			
WHR	−0.07	−0.17	−0.18			
Systolic BP	0.20 *	0.19	0.03			
Diastolic BP	0.33 **	0.30 *	0.20			
Total Cholesterol	−0.06	−0.08	−0.03	0.04	0.03	0.10
HDL-C	−0.30 **	0.11	−0.37 **	−0.06	0.22	−0.10
LDL-C	−0.05	−0.18	0.02	0.02	0.14	0.01
Triglycerides	0.10	−0.03	0.14	0.12	−0.19	0.23 *
25(OH)D	−0.04	0.11	−0.16	−0.17 *	−0.27 *	−0.16
Glucose	0.13	0.32 *	0.04	0.12	0.04	0.14
CRP	0.55 **	0.54 **	0.55 **	0.39 **	0.61 **	0.47 **

Note: Data presented as coefficient (R); * denotes significance at 0.05 level; ** denotes significance at 0.01 level.

**Table 5:** Delta change associations between study parameters among responders.

All Participants								
	Δ SAP	Δ CRP	Δ Cholesterol	Δ HDL	Δ LDL	Δ Triglycerides	Δ VD	Δ Glucose
Δ SAP	1.00							
Δ CRP	0.28 **	1.00						
Δ TC	−0.05	0.26 *	1.00					
Δ HDL-C	−0.30 **	−0.16	0.33 **	1.00				
Δ LDL-C	0.11	0.31 **	0.75 **	−0.01	1.00			
Δ Triglycerides	−0.05	0.05	0.29 **	−0.06	−0.15	1.00		
Δ 25(OH)D	0.01	−0.12	−0.04	0.15	0.00	−0.10	1.00	
Δ Glucose	0.06	0.34 **	0.19 *	−0.09	0.15	0.16	−0.19 *	1.00
**Males**								
Δ SAP	1.00							
Δ CRP	0.11	1.00						
Δ TC	−0.07	0.17	1.00					
Δ HDL-C	−0.31 *	0.10	0.00	1.00				
Δ LDL-C	0.01	0.31	0.83 **	0.17	1.00			
Δ Triglycerides	−0.27 *	−0.11	0.25	−0.18	−0.24	1.00		
Δ 25(OH)D	−0.02	−0.13	−0.21	0.15	−0.25	0.00	1.00	
Δ Glucose	0.22	0.06	−0.11	−0.04	−0.08	−0.09	−0.05	1.00
**Females**								
Δ SAP	1.00							
Δ CRP	0.09	1.00						
Δ TC	−0.06	0.08	1.00					
Δ HDL-C	−0.16	−0.13	0.29 **	1.00				
Δ LDL-C	0.02	0.06	0.71 **	−0.04	1.00			
Δ Triglycerides	−0.07	0.26	0.42 **	−0.05	0.10	1.00		
Δ 25(OH)D	0.10	−0.03	0.07	0.00	0.02	−0.02	1.00	
Δ Glucose	−0.12	0.07	0.25 *	−0.17	0.25 *	0.13	−0.14	1.00

Note: Data presented as coefficient (R); * denotes significance at 0.05 level; ** denotes significance at 0.01 level. VD: Vitamin D.

**Table 6:** Clinical characteristics of responders at baseline and after 6-month intervention using SAP cut-off levels.

Parameters	Male (SAP > 30 mg/L)					Female (SAP > 24 mg/L)				
	Baseline	6-Month	Δ	Effect Size	*p*-Value	Baseline	6-Month	Δ	Effect Size	*p*-Value
N (M/F)	42					56				
Age (year)	41.4 ± 8.8					38.8 ± 11.6				
BMI (kg/m^2^)	27.7 ± 3.0					29.8 ± 4.4				
WHR	0.98 ± 0.06					0.89 ± 0.10				
SBP (mmHg)	132.0 ± 11.2					121.5 ± 13.4				
DBP (mmHg)	82.6 ± 6.7					76.8 ± 8.4				
T. Chol (mmol/L)	4.9 ± 1.1	5.0 ± 1.4	0.1 (−0.1–0.3)	0.12	0.41	5.2 ± 1.3	5.2 ± 1.2	0.03 (−0.3–3)	0.026	0.85
HDL-C (mmol/L)	0.97 ± 0.3	1.1 ± 0.3	0.1 (0.05–0.2)	0.52	0.002	1.0 ± 0.4	1.2 ± 0.5	0.21 (0.05–0.4)	0.36	0.01
LDL-C (mmol/L)	3.1 ± 0.8	3.0 ± 0.9	−0.08 (−0.3–0.2)	0.10	0.51	3.3 ± 0.9	3.1 ± 1.0	−0.12 (−0.4–0.1)	0.13	0.32
TG (mmol/L)	0.03 ± 0.8	0.15 ± 0.5	0.1 (−0.05–0.3)	0.21	0.16	0.04 ± 1.1	0.21 ± 0.8	0.17 (0.03–0.3)	0.34	0.016
VD (nmol/L)	34.9 ± 9.7	63 ± 14.1	28 (23–33)	1.61	<0.001	28.6 ± 11.4	59.6 ± 20.7	30.9 (26–36)	1.53	<0.001
Glucose (mmol/L)	5.8 ± 0.9	5.9 ± 0.9	0.14 (−0.1–0.4)	0.21	0.25	5.5 ± 1.1	5.4 ± 0.9	−0.06 (−0.4–0.3)	0.06	0.71
SAP (mg/L)	82 (53–109)	65 (44–88)	−9.5 (−34–7)	0.39	0.007	64 (46–104)	58.4 (38–76)	−13.9 (−33–2.2)	0.56	<0.001
CRP (µg/mL)	36.2 (9–49)	27.5 (10–41)	−3.0 (−16–4)	0.32	0.07	27.7 (5–62)	9.1 (5.1–33)	−0.4 (−30.1–3.6)	0.33	0.038

Note: Data presented as mean ± SD for normal and median (first–third) percentiles for non-normal variables. Significant at *p* < 0.05.

## References

[1] Vanchinathan V., Lim H.W. (2012). A dermatologist’s perspective on vitamin D. Mayo Clin. Proc..

[2] Lee J.H., O’Keefe J.H., Bell D., Hensrud D.D., Holick M.F. (2008). Vitamin D deficiency an important, common, and easily treatable cardiovascular risk factor?. J. Am. Coll. Cardiol..

[3] Pfeifer M., Begerow B., Minne H.W. (2002). Vitamin D and muscle function. Osteoporos. Int..

[4] Heaney R.P. (2008). Vitamin D in health and disease. Clin. J. Am. Soc. Nephrol..

[5] Cohen-Lahav M., Douvdevani A., Chaimovitz C., Shany S. (2007). The anti-inflammatory activity of 1,25-dihydroxyvitamin D3 in macrophages. J. Steroid Biochem. Mol. Biol..

[6] Gysemans C.A., Cardozo A.K., Callewaert H., Giulietti A., Hulshagen L., Bouillon R., Eizirik D.L., Mathieu C. (2005). 1,25-Dihydroxyvitamin D3 modulates expression of chemokines and cytokines in pancreatic islets: Implications for prevention of diabetes in nonobese diabetic mice. Endocrinology.

[7] Hossein-Nezhad A., Mirzaei K., Keshavarz S.A., Ansar H., Saboori S., Tootee A. (2013). Evidences of dual role of vitamin D through cellular energy homeostasis and inflammation pathway in risk of cancer in obese subjects. Minerva Med..

[8] Calton E.K., Keane K.N., Newsholme P., Soares M.J. (2015). The Impact of Vitamin D Levels on Inflammatory Status: A Systematic Review of Immune Cell Studies. PLoS ONE.

[9] Prietl B., Treiber G., Pieber T.R., Amrein K. (2013). Vitamin D and immune function. Nutrients.

[10] Chen N., Wan Z., Han S.-F., Li B.-Y., Zhang Z.-L., Qin L.-Q. (2014). Effect of vitamin D supplementation on the level of circulating high-sensitivity C-reactive protein: A meta-analysis of randomized controlled trials. Nutrients.

[11] Calton E.K., Keane K.N., Soares M.J. (2015). The potential regulatory role of vitamin D in the bioenergetics of inflammation. Curr. Opin. Clin. Nutr. Metab. Care.

[12] Rodriguez A.J., Mousa A., Ebeling P.R., Scott D., de Courten B. (2018). Effects of vitamin D supplementation on inflammatory markers in heart failure: A systematic review and meta-analysis of randomized controlled trials. Sci. Rep..

[13] Christner R.B., Mortensen R.F. (1994). Specificity of the binding interaction between human serum amyloid P-component and immobilized human C-reactive protein. J. Biol. Chem..

[14] Lu J., Marjon K.D., Mold C., Du Clos T.W., Sun P.D. (2012). Pentraxins and Fc receptors. Immunol. Rev..

[15] Deban L., Bottazzi B., Garlanda C., de la Torre Y.M., Mantovani A. (2009). Pentraxins: Multifunctional proteins at the interface of innate immunity and inflammation. Biofactors.

[16] Steel D.M., Whitehead A.S. (1994). The major acute phase reactants: C-reactive protein, serum amyloid P component and serum amyloid A protein. Immunol. Today.

[17] Ravnsborg T., Svaneklink S., Andersen L.L.T., Larsen M.R., Jensen D.M., Overgaard M. (2019). First-trimester proteomic profiling identifies novel predictors of gestational diabetes mellitus. PLoS ONE.

[18] Maekawa Y., Nagai T., Anzai A. (2011). Pentraxins: CRP and PTX3 and cardiovascular disease. Inflamm. Allergy Drug Targets.

[19] Al-Daghri N.M., Al-Attas O.S., Wani K., Alnaami A.M., Sabico S., Al-Ajlan A., Chrousos G.P., Alokail M.S. (2015). Sensitivity of various adiposity indices in identifying cardiometabolic diseases in Arab adults. Cardiovasc. Diabetol..

[20] Al-Daghri N.M., Al-Othman A., Albanyan A., Al-Attas O.S., Alokail M.S., Sabico S., Chrousos G.P. (2014). Perceived stress scores among Saudi students entering universities: A prospective study during the first year of university life. Int. J. Environ Res. Public Health.

[21] Al-Daghri N.M., Al-Saleh Y., Aljohani N., Sulimani R., Al-Othman A.M., Alfawaz H., Fouda M., Al-Amri F., Shahrani A., Alharbi M. (2017). Vitamin D status correction in Saudi Arabia: An experts’ consensus under the auspices of the European Society for Clinical and Economic Aspects of Osteoporosis, Osteoarthritis, and Musculoskeletal Diseases (ESCEO). Arch. Osteoporos..

[22] Al Saleh Y., Beshyah S.A., Hussein W., Almadani A., Hassoun A., Al Mamari A., Ba-Essa E., Al-Dhafiri E., Hassanein M., Fouda M.A. (2020). Diagnosis and management of vitamin D deficiency in the Gulf Cooperative Council (GCC) countries: An expert consensus summary statement from the GCC vitamin D advisory board. Arch. Osteoporos..

[23] Nelson S.R., Tennent G.A., Sethi D., Gower P.E., Ballardie F.W., Amatayakul-Chantler S., Pepys M.B. (1991). Serum amyloid P component in chronic renal failure and dialysis. Clin. Chim. Acta.

[24] Bottazzi B., Inforzato A., Messa M., Barbagallo M., Magrini E., Garlanda C., Mantovani A. (2016). The pentraxins PTX3 and SAP in innate immunity, regulation of inflammation and tissue remodelling. J. Hepatol..

[25] Emsley J., White H.E., O’hara B.P., Oliva G., Srinivasan N., Tickle I.J., Blundell T.L., Pepys M.B., Wood S.P. (1994). Structure of pentameric human serum amyloid P component. Nature.

[26] Ashton A.W., Boehm M.K., Gallimore J.R., Pepys M.B., Perkins S.J. (1997). Pentameric and decameric structures in solution of serum amyloid P component by X-ray and neutron scattering and molecular modelling analyses. J. Mol. Biol..

[27] Li X.A., Yutani C., Shimokado K. (1998). Serum amyloid P component associates with high density lipoprotein as well as very low density lipoprotein but not with low density lipoprotein. Biochem. Biophys. Res. Commun..

[28] Li X.A., Hatanaka K., Ishibashi-Ueda H., Yutani C., Yamamoto A. (1995). Characterization of serum amyloid P component from human aortic atherosclerotic lesions. Arterioscler. Thromb. Vasc. Biol..

[29] Cubedo J., Padró T., Badimon L. (2013). Coordinated proteomic signature changes in immune response and complement proteins in acute myocardial infarction: The implication of serum amyloid P-component. Int. J. Cardiol..

[30] Zheng L., Wu T., Zeng C., Li X., Li X., Wen D., Ji T., Lan T., Xing L., Li J. (2016). SAP deficiency mitigated atherosclerotic lesions in ApoE(-/-) mice. Atherosclerosis.

[31] Xi D., Luo T., Xiong H., Liu J., Lu H., Li M., Hou Y., Guo Z. (2015). SAP: Structure, function, and its roles in immune-related diseases. Int. J. Cardiol..

[32] Ogawa T., Kawano Y., Imamura T., Kawakita K., Sagara M., Matsuo T., Kakitsubata Y., Ishikawa T., Kitamura K., Hatakeyama K. (2010). Reciprocal contribution of pentraxin 3 and C-reactive protein to obesity and metabolic syndrome. Obesity.

[33] Al-Daghri N.M., Al-Attas O.S., Johnston H.E., Singhania A., Alokail M.S., Alkharfy K.M., Abd-Alrahman S.H., Sabico S.L., Roumeliotis T.I., Manousopoulou-Garbis A. (2014). Whole serum 3D LC-nESI-FTMS quantitative proteomics reveals sexual dimorphism in the milieu intérieur of overweight and obese adults. J. Proteome Res..

[34] Al-Daghri N.M., Mohammed A.K., Bukhari I., Rikli M., Abdi S., Ansari M.G.A., Sabico S., Hussain S.D., Alenad A., Al-Saleh Y. (2019). Efficacy of vitamin D supplementation according to vitamin D-binding protein polymorphisms. Nutrition.

